# Déterminants de la calcémie néonatale dans une maternité périphérique à Abidjan

**DOI:** 10.11604/pamj.2015.20.390.6138

**Published:** 2015-04-21

**Authors:** Diby Jean-Paul Konan, Flore Amon-Tanoh-Dick, Joseph Aka, Sess Essiagne Daniel

**Affiliations:** 1 Laboratoire de Biostatistique et d'Informatique Médicale, UFR Sciences Médicales - Université Félix Houphouët-Boigny, Abidjan, Côte d'Ivoire; 2 Service de Néonatalogie - CHU Yopougon, Côte d'Ivoire; 3 Institut National de Santé Publique, Côte d'Ivoire; 4 Laboratoire de Biochimie, UFR Sciences Médicales, Abidjan, Côte d'Ivoire

**Keywords:** Hypocalcémie, accouchement, nouveau-né, parturiente, Côte d´Ivoire, Hypocalcemia, delivery, newborn, parturient, Ivory Coast

## Abstract

**Introduction:**

Le transfert du calcium de la mère au nouveau-né est nécessaire pour la constitution de la charpente osseuse. L'hypocalcémie néonatale reste peu documentée en Afrique. En 1993, il a été observé au service de néonatologie du Centre hospitalo-universitaire de Yopougon à Abidjan une proportion d'hypocalcémie asymptomatique de 26.9%. L'objectif de ce travail était de préciser la prévalence de l'hypocalcémie néonatale dans une maternité périphérique de Yopougon et en déterminer les facteurs de risque.

**Méthodes:**

Une étude transversale a été menée à la formation sanitaire de de Yopougon de février à mai 2012 auprès de 145 parturientes et leurs nouveau-nés. Les caractéristiques des mères et des nouveau-nés ont été recueillies. Les prélèvements de sang maternel et du cordon ont permis le dosage des paramètres du métabolisme phosphocalcique. Une régression linéaire multiple a été faite pour apprécier les éléments de prédilection de la calcémie néonatale.

**Résultats:**

Les valeurs moyennes étaient de 2,271 mmol/l pour la calcémie, 1,169 mmol/l pour le phosphore, 0,735 mmol/l pour le magnésium et 69 g/l pour les protides totaux chez les mères. La durée moyenne du travail était de 499 minutes. L’âge gestationnel moyen était de 39 semaines. Les constantes anthropométriques et cliniques des nouveau-nés étaient normales. Les paramètres phosphocalciques étaient en moyenne normaux (calcémie = 2,52 mmol/l, phosphorémie = 1,668 mmol/l, magnesémie = 0,777 mmol/l, protidémie = 63 g/l). Six gestantes (4,1%) étaient hypocalcémiques contre 12 nouveau-nés (8,1%). La durée du travail influençait la calcémie néonatale (p = 0,02). En analyse multivariée, 34% de la variabilité de la calcémie néonatale était expliqué par la calcémie maternelle et la durée du travail.

**Conclusion:**

L'hypocalcémie néonatale est rare en zone tropicale. Dans cette étude, la proportion était de 8,1%. La durée du travail influençait la calcémie néonatale. Aussi, nous recommandons de poursuivre les investigations en dosant, en plus, la vitamine D et le Parathormone de la naissance à J7 de vie et préciser les étiologies de ces hypocalcémies.

## Introduction

Le calcium est un élément minéral important dans l'organisme. Bien que la plupart du calcium dans le corps se trouve dans les os et les dents. Mais moins de 1% a des fonctions critiques de maintien de la vie [1, 2]. Au niveau de l'organisme, il est intimement lié à de nombreux processus biologiques vitaux [3]. A ce titre, il intervient dans diverses fonctions. La principale fonction du calcium, en combinaison avec le phosphore est de fournir une structure rigide au système squelettique humain. En outre, il intervient dans la fonction des membranes cellulaires et la régulation du métabolisme cellulaire, dans la coagulation sanguine, dans la transmission de l'influx nerveux et la concentration musculaire. Au cours de la grossesse, le but de l´homéostasie du calcium de la mère est de fournir un flux de calcium adéquat à travers le placenta pendant la grossesse et dans le lait maternel pendant l´allaitement, afin d´assurer une minéralisation normale du squelette fœtal et néonatal [2, 4]. La minéralisation du squelette fœtal qui se fait tout au long de la grossesse a lieu principalement au 3^ème^ trimestre puisque 80% du transfert s'effectue à cet âge gestationnel [5, 6]. En zone tempérée, l'hypocalcémie néonatale est bien documentée [7–10]. En Afrique subsaharienne, ce tableau est rare et est décrit en cas de diabète, d'accouchement dystocique ou en cas de déficit en Vitamine D. Ce tableau clinique est dominé par les signes neurologiques avec leurs conséquences sur le développement neurologique ultérieur de l'enfant [11]. Dans cette zone, l'hypocalcémie néonatale est souvent asymptomatique et méconnue lorsqu'elle est modérée Ainsi, en 2005, une étude, non publiée sur le calcium et les pathologies néonatales en milieu hospitalier dans le service de néonatologie du CHU de Yopougon, a révélé une prévalence d'hypocalcémie asymptomatique de 26.9%. Les nouveau-nés seraient-ils hypocalcémiques à leur naissance? Aussi, cette étude visait à préciser la prévalence de l'hypocalcémie néonatale dans la maternité périphérique de Yopougon et en déterminer les facteurs de risque.

## Méthodes

L’étude s'est effectuée dans la maternité de la Formation Sanitaire à base Communautaire de Yopougon Toit-Rouge (FSUCOM-YOP) dans la commune de Yopougon. C'est une structure de santé de premier contact situé dans un quartier à forte densité. La structure de référence à proximité est le Centre Hospitalier Universitaire de Yopougon. Il s'agissait d'une étude transversale qui s'est déroulée à la maternité du centre de santé du 21 février au 18 mai 2012 sur un échantillon de 145 parturientes. Toute gestante, en travail, venue dans le centre pour accoucher et ayant acceptée d’être prélevée, après le consentement éclairé, a été incluse. Ont été exclues les gestantes ayant reçu une médication à base de calcium et/ou de magnésium au cours de la grossesse ainsi que celle ayant reçu une médication à base de magnésium au cours du travail. Ont également été exclues, toutes gestantes présentant un diabète gestationnel ou une maladie affectant les parathyroïdes. Au total 145 parturientes et leurs nouveau-nés ont été inclus dans l’étude. Une fiche d'enquête a permis le recueil des données sur les caractéristiques sociodémographiques et obstétricales de la parturiente et les données anthropométrique et cliniques du nouveau-né. Pour le dosage des paramètres du métabolisme phosphocalcique, des prélèvements ont été effectués sur tube sec. Pour le nouveau-né, le sang du cordon a été prélevé juste après la ligature du cordon et asepsie rigoureuse avec de l'alcool à 60°. Quant aux mères, le prélèvement a été effectué, juste après l'accouchement avant la délivrance, au pli du coude. Les échantillons ont été placés dans une glacière et acheminés au laboratoire de biochimie du Centre Hospitalier Universitaire (CHU) de Yopougon. Ils ont été centrifugés et le dosage des différents paramètres a été effectué sur un automate. Les paramètres dosés étaient: la calcémie, la phosphorémie, la magnésémie et la protidémie. La vitamine D n'a pas été dosée car le déficit en vitamine D est rare dans la zone tropicale. En effet la Vitamine D est synthétisée à 95% par le soleil [12, 13] La classification des valeurs des paramètres dosées s'est faite à partir des valeurs rapportées dans les données de la littérature [14–16]. Les valeurs seuil pour confirmer le déficit d'un paramètre sanguin ont été définies selon différents auteurs [6, 17–19].

Ainsi, pour le nouveau-né, l'hypocalcémie était définie par une calcémie < 2,25 mmol/L et au-delà il a été considéré comme normale. Pour la phosphorémie et magnésémie, les valeurs seuil étaient respectivement de 1,292 mmol/l et de 0,600 mmol/l [17]. Quant à la protidémie, le déficit était décidé à partir d'une valeur inférieure à 50 mg/l. Pour la mère, l'hypocalcémie était définie à partir d'une valeur seuil de 2 mmol/L. Pour apprécier la trophicité, le percentile exact pour le poids de naissance et l’âge gestationnel, de chaque nouveau-né, a été déterminé, La saisie des données a été faite avec le logiciel EpiData Version 3.1 et l'analyse a été effectuée avec le logiciel R. L'analyse a été descriptive avec calcul des paramètres de tendance centrale et de dispersion pour les variables quantitatives et de proportion pour les variables qualitatives. Le Test de KHI2 a été utilisé pour comparer les proportions et le test T de Student pour comparer les moyennes. Pour apprécier la relation entre la calcémie maternelle et néonatale une régression linéaire a été effectuée. Pour déterminer ce qui prédit le mieux la calcémie du nouveau-né, une régression linéaire multiple a été effectuée. La variable dépendante était la calcémie néonatale, la variable d'intérêt primaire était la calcémie maternelle. Le seuil de signification des tests statistiques utilisés était fixé à 5%.

## Résultats

Au cours des 3 mois de l’étude, 145 gestantes et leurs nouveau-nés ont été incluses. Dans notre échantillon, l’âge moyen des mères étaient de 26 ans. Elles avaient au moment de l'accouchement une hauteur utérine moyenne de 33 cm et la durée moyenne du travail était de 500 minutes avec de grandes variations (écart-type de 225 minutes). Elles avaient pris au moment de l'accouchement un moyen de 10 Kg (Tableau 1). Pour les nouveau-nés, ils étaient tous à terme avec un âge gestationnel moyen de 39 semaines d'aménorrhée. Ils avaient les constantes anthropométriques normales (poids moyen = 3 000 g, taille moyenne = 50 cm, périmètre crânien = 33 cm). Leur état neurologique était normal avec un score d'Apgar moyen à 1 minute de 7 (Tableau 1). Les paramètres phosphocalciques des mères étaient en moyenne de 2,271 mmol/l pour la calcémie, 1,169 mmol/l pour le phosphore, 0,735 mmol/l pour le magnésium et 69 g/l pour les protides totaux. Les valeurs moyennes chez le nouveau-nés étaient pour la calcémie de 2,52 mmol/l, la phosphorémie de 1,668 mmol/l, la magnésémie de 0,777 mmol/l et la protidémie = 63 g/l) (voir Tableau 2). En analyse bivariée, on notait une différence statistiquement significative entre les paramètres phosphocalciques chez les nouveau-nés et leurs mères (p < 0.001). On observait un gradient positif entre les paramètres des nouveau-nés et des mères sauf pour la protidémie (p < 0,01) (Tableau 2). A la naissance, il y avait six (6) gestantes (4,1%) hypocalcémiques et la prévalence de l'hypocalcémie néonatale était de 8,1%. On retrouvait des proportions d'hypomagnésémie de 37% chez les gestantes et 4,8% pour les nouveau-nés (Tableau 3). Il n'y avait pas d'association entre la trophicité, le poids de naissance et la calcémie du nouveau-né. Il en était de même pour la présence de signe d'asphyxie (Tableau 4). Chez les mères, la prise de poids, et l’âge maternel n’était pas associés à la calcémie maternelle (Tableau 5). Par contre la durée du travail influençait sur la calcémie néonatale et maternelle. La différence était statistiquement significative (Tableau 4, Tableau 5). Pour apprécier la prédiction de la calcémie néonatale, une régression linéaire multiple a été effectuée. La variable dépendante était la calcémie néonatale, la variable d'intérêt primaire était la calcémie maternelle. D'autres variables (magnesémie, phosphorémie, protidémie maternelle, poids de naissance, Apgar, terme de la grossesse et durée du travail) ont été ajoutées au modèle. Par l'analyse pas à pas descente, la calcémie maternelle et la durée du travail étaient significatives. Le modèle final est donc une régression linéaire multiple avec la calcémie néonatale comme variable dépendante, la calcémie maternelle et la durée du travail étaient les variables prédicatrices (Tableau 6). Près de 34% de la variabilité de la calcémie néonatale est expliqué par une relation linéaire multiple avec la calcémie maternelle et la durée du travail.

**Tableau 1:** Caractéristiques des parturientes et des nouveau-nés

Paramètres (n = 145)	Moyenne (Ecart-type)
Mère	
Age de la Mère (ans)	25,9 (5,3)
Prise de poids (Kg)	9,5 (2,7)
Hauteur utérine (cm)	33,0 (1, 5)
Durée du travail (en minutes)	499,3 (225,1)
Nouveau-nés	
Age gestationnel (semaines)	39,2 (1,4)
Poids de naissance (grammes)	3 072 (381)
Trophicité poids/âge gestationnel (percentile)	34,8 (23,9)
Taille de naissance (cm)	49,8 (1,8)
Périmètre crânien (cm)	33,1 (2,5)
Apgar à 5 min	8,5 (0,8)
Fréquence respiratoire (cycles/min)	44,6 (6,3)
Fréquence cardiaque (battements/min)	141,1 (7,2)

**Tableau 2:** Paramètres phosphocalciques des parturientes et des nouveau-nés

Paramètres phosphocalciques	Moyenne (Ecart-type)		Médiane		P	Test de corrélation	
	(valeurs normales)		(Min - Max)		
	Mères^1^	Nouveau-nés^2^	Mères	Nouveau-nés		R^2^	p
Calcémie (mmol/L)	2, 271 (0,172)	2,520 (0,174)	2,253	2,557	<0,001	0,314	<0,001
	(2-2,5)	(2,25-2,875)	(1,780-2,629)	(2,054-2,951)			
Phosphorémie (mmol/L)	1,169 (0,300)	1,668 (0,422)	1,134	1,625	<0,001	0,268	<0,001
	(0,969-1,454)	(1,292-2,584)	(0,291-2,125)	(0,791-3,198)			
Magnésémie (mmol/L)	0,735 (0,109)	0,777 (0,120)	0,727	0,7645	0,001	0,060	0,003
	(0,740-0,904)	(0,863-1,027)	(0,370-1,052)	(0,383-1,229)			
Protidémie (g/l)	68,6 (7,4)	62,5 (7,5)	68,30	62	<0,001	0,040	0,011
	(55-65)	(50-75)	(39,4-87,6)	(38-81,3)			

1 Les valeurs de référence sont issues de la référence [14, 17]

2 Les valeurs de référence sont tirées du livre « Pédiatrie » de Yannick Aujard, référence [16, 17]

**Tableau 3:** Déficits minéraux des parturientes et des nouveau-nés

Paramètres	Effectifs	Pourcentage (%)
Mère (n = 145)		
Hypocalcémie	6	4,1
Hypomagnésémie	54	37,2
Hypophosphorémie	35	24,1
Hypoprotidémie	6	4,1
Nouveau-né (n = 145)		
Hypocalcémie	12	8,1
Hypomagnésémie	7	4,8
Hypophosphorémie	22	15,2
Hypoprotidémie	6	4,1

**Tableau 4:** Calcémie néonatale et caractéristiques du nouveau-né

Caractéristiques des nouveau-nés (n = 145)	Effectif	Calcémie moyenne(Ecart-type)	p
Trophicité en percentile (Poids de naissance et âge gestationnel)			
Inférieur à 3%	9	2, 271 (0,179)	0,999
Supérieur 3%	136	2,271 (0,172)	
Poids de naissance			
Inférieur à 2500 g	11	2,427(0,162)	0,068
A partir de 2500 g	134	2,526 (0,173)	
Présence d'une asphyxie (APGAR à 5 min < 7)			
Non	142	2,520 (0,173)	0,550
Oui	3	2,459 (0,242)	
Durée du travail			
Inférieure à 8 H	72	2,563 (0,1547)	0,002
Supérieure ou égal à 8 H	73	2,475 (0,182)	

**Tableau 5:** Calcémie de la parturiente et caractéristiques maternelles

Caractéristiques maternels (n = 145)	Effectif	Calcémie moyenne (Ecart-type)	p
Durée du travail			
Inférieure à 8 H	72	2,300 (0,164)	0,035
Supérieure ou égal à 8 H	73	2,243 (0,176)	
Prise de Poids			
Moins de 12 Kg	119	2,269 (1,673)	0,794
12 Kg et Plus	26	2,279 (1,936)	
Age de la mère			
Moins de 25 ans	61	2,267 (1,779)	0,810
25 ans et Plus	84	2,273 (1,680)	

**Tableau 6:** Calcémie néonatale ajustée pour la calcémie maternelle et la durée du travail

Variables	Coefficient	p	R2
Calcémie maternelle	0,567	0,000	0,340
Durée du travail	-0,007	0,021	
Constante	1,294	0,000	

## Discussion

Cette étude a analysé les prélèvements sanguins effectués chez les mères à l'accouchement et le sang du cordon pour leurs nouveau-nés. Elle n'a pas pris en compte le sang des nouveau-nés à 2-4 jours de vie, période au cours de laquelle l'hypocalcémie est la plus fréquente [9, 18–21]. L’âge moyen était de 25,9 ans (écart-type de 5,3). Nos résultats reflètent le profil des femmes ayant une fécondité maximale et est similaire à ceux de l'Enquête Démographique et de Santé (EDS) en Côte d'Ivoire 1998-1999 [22]. En effet l'EDS avait trouvé que la tranche d’âge de 25-29 ans était la plus féconde. Notre étude a montré une différence significative entre la calcémie maternelle et néonatale. Cette observation a été également faite par Pitkin R.M et al [23] et Salle B et Perelman R [24] qui ont trouvé des valeurs de 2,75 mmol/l et 2,3 mmol/l pour respectivement la calcémie néonatale et maternelle [5, 6, 14, 23, 25–27]. Cette différence s'expliquerait par le fait qu'au cours du dernier trimestre de la grossesse, il y a une chute importante de la calcémie maternelle. En effet, pendant la grossesse, il y a un mécanisme assure le transfert de la calcémie maternelle vers le fœtus pour constituer sa charpente squelettique [9, 14, 15]. Toutes les caractéristiques des mères et de leurs nouveau-nés sont normales. Nous pensons que cet état se justifie par le fait que l’étude s’étant déroulé dans un centre de santé périphérique, il n'y avait pas de cas de complications. Car les cas de complications, comme les accouchements dystociques étaient dirigés vers le centre de référence. Parmi les 12 nouveau-nés hypocalcémiques, aucun n'avait de signes évidents d'hypocalcémie. Les signes observés sont des signes d'asphyxie. Cette absence, de signes évidents, serait probablement due au fait que la calcémie à la naissance est élevée (supérieur à 2,5 mmol/l) [10, 18, 28]. Dans notre étude, la calcémie cordonale moyenne était supérieure à 2,5 mmol/L. En effet, la calcémie néonatale évolue peu avant les 48H. La présence de signes, évidents d'hypocalcémie, se voit chez les nouveau-nés à haut risque. Il s'agit, dans ce cas, de nouveau-nés de mères diabétiques, de mères ayant eu une thyroïdectomie ou para thyroïdectomie [10, 11, 28, 29].

Or les nouveau-nés étaient apparemment sains sans signes de complications. Dans notre étude cinq (3,4%) parturientes et 12 (8,1%) nouveau-nés avaient une hypocalcémique. La différence avec les données personnelles, non publiées avec une proportion de 26,9% de nouveau-nés hypocalcémiques, s'expliquerait par le fait que la population d’étude était constituée de nouveau-nés pathologiques résultants de grossesse à risque et de mode d'accouchement à risque. Les données sur la calcémie maternelle de notre étude étaient similaires à d'autres études [23, 26, 27]. Cependant, elle est différente des résultats de Akpona SA et al. au Bénin qui ont trouvé une calcémie maternelle de 1,778 ± 0,425 mmol/L [25]. Ces différences pourraient s'expliquer par les périodes de prélèvement. Dans l’étude réalisée au Bénin, le calcium maternel a été dosé avant l'accouchement et dans le post-partum chez des parturientes allaitantes. Les résultats des autres paramètres (phosphore, magnésium et protides) étaient comparables aux données de la littérature Nos résultats pour les autres paramètres (phosphore, magnésium et protides) étaient comparables aux données de la littérature [2, 9, 14, 23–25]. En comparant la calcémie néonatale avec certaines caractéristiques du nouveau-né comme le poids à la naissance et la présence de souffrance cérébrale, nous avions constaté qu'il n'y avait pas de différence significative dans les différentes classes. Cela s'expliquerait par le fait que la majorité des nouveau-nés sont normaux et tous les accouchements compliqués sont référencés. Par contre la durée du travail influençait de manière significative la calcémie maternelle et néonatale. En effet, la durée du travail contribue à la baisse de la calcémie. Dans la mesure où le calcium est vital dans le mécanisme de la contraction utérine [2, 3, 14, 21, 30].

## Conclusion

Cette étude montre que la calcémie des nouveau-nés et de leurs mères étaient normales en moyenne au moment de l'accouchement. Cependant, la durée du travail influençait la calcémie néonatale. Dans la littérature, l'hypocalcémie néonatale est rare en zone tropicale. Or le mode de vie, en particulier vestimentaire, pourrait influencer ces paramètres biologiques. Compte tenu des conséquences d'une hypocalcémie néonatale sur le devenir neurologique du nouveau-né, nous recommandons de poursuivre les investigations, dans les mêmes conditions, en dosant la vitamine D et le Parathormone de la naissance à J7 de vie. Ceci permettrait d’établir la cinétique de la calcémie néonatale en zone tropicale et prenant en compte le mode de vie. Par ailleurs, un suivi clinique et biologique des nouveau-nés avec une hypocalcémie au cordon permettrait de déterminer l’étiologie et le devenir de ces nouveau-nés. Ces travaux permettraient d'envisager ultérieurement un dosage de la calcémie chez les nouveau-nés en cas d'accouchements prolongés.
